# Re-emergence of diphtheria in Germany: a national population-based surveillance study of *Corynebacterium diphtheriae* and *Corynebacterium ulcerans*, 2017 to 2025

**DOI:** 10.2807/1560-7917.ES.2026.31.45.2600348

**Published:** 2026-11-12

**Authors:** Lena Ella Schneider, Franziska Badenschier, Katja Bengs, Mario Martín-Sánchez, Christiane Wagner-Wiening, Wiebe Külper-Schiek, Ole Wichmann, Anja Berger, Andreas Sing, Cornelius Rau, Delphine Perriat

**Affiliations:** 1 Department for Infectious Disease Epidemiology, Robert Koch Institute, Berlin, Germany; 2 Postgraduate Training for Applied Epidemiology, Department for Infectious Disease Epidemiology, Robert Koch Institute, Berlin, Germany; 3 ECDC Fellowship Programme, Field Epidemiology path (EPIET), European Centre for Disease Prevention and Control (ECDC), Stockholm, Sweden; 4 National Conciliary Laboratory for Diphtheria, Bavarian Health and Food Safety Authority, Oberschleißheim, Germany; 5 EU Public Health Reference Laboratory for Diphtheria and Pertussis (EURL-PH-EUDIPE), Oberschleißheim, Germany; 6 WHO Collaborating Centre for Diphtheria, Oberschleißheim, Germany; 7 Department of Health Protection, Infection Control and Epidemiology, State Health Office, Ministry of Social Affairs, Health and Integration, Stuttgart, Germany

**Keywords:** Corynebacterium diphtheriae, Diphtheria, Disease outbreaks, Migration and health, Vaccine-preventable disease

## Abstract

**BACKGROUND:**

Diphtheria is a rare vaccine-preventable disease in Germany, but since 2022 *Corynebacterium diphtheriae* cases have increased following an outbreak among newly arriving migrants.

**AIM:**

We described epidemiological trends and differences between *C. diphtheriae* and *C. ulcerans* cases.

**METHODS:**

We included laboratory-confirmed (toxigenic or *tox* gene positive) diphtheria cases and cases epidemiologically linked to laboratory-confirmed cases in Germany in 2017–2025. Enhanced surveillance data were available for a subset of cases in 2017–2022. We analysed cases by species assessing clinical, demographic and epidemiological characteristics descriptively, with chi-square and Fisher’s exact tests.

**RESULTS:**

Overall, 512 diphtheria cases were reported (346 *C. diphtheriae*; 166 *C. ulcerans*; incidence: 0.07/100,000 population) including five deaths (three *C. diphtheriae*, two *C. ulcerans*, all reported since 2022). Of these, 460 (90%) cases had cutaneous, 30 (6%) respiratory and 22 (4%) double manifestation. Four epidemiological phases were identified. Acquisition of *C. diphtheriae* shifted from predominantly migration-associated (232/261) in 2022–2023 to autochthonous transmission (35/54) in 2024–2025. Autochthonous *C. diphtheriae* cases were older and more often hospitalised than imported cases (≥ 45 years: 23/45 vs 19/283; p < 0.001; hospitalisation: 36/45 vs 83/283; p < 0.001). All *C. ulcerans* cases were autochthonous and 70 of 89 (79%) reported contact with companion animals.

**CONCLUSION:**

Diphtheria is re-emerging in Germany. Double manifestations warrant attention, being almost as common as respiratory manifestations. Clinicians should be aware of the disease to facilitate prompt diagnosis and treatment. Preventing severe cases and further transmission requires strengthening routine and catch-up vaccination services, particularly for older individuals and people experiencing homelessness.

**Key public health message**

**What did you want to address in this study and why?**

Diphtheria can present as a severe respiratory and/or skin disease that can be prevented by vaccination. Only a few persons are reported with diphtheria in Germany every year, but the numbers have increased since 2022. Mainly two bacterial species, *Corynebacterium diphtheriae* and *Corynebacterium ulcerans*, cause diphtheria, and we wanted to better understand how the dynamics of these infections have changed over time.

**What have we learnt from this study?**

Between 2017 and 2025, 512 patients with diphtheria were reported in Germany, 68% were caused by *C. diphtheriae*. Most patients (90%) had a skin infection. In 2017–2023, most patients with *C. diphtheriae* were infected abroad through travel or migration. Patients with *C. ulcerans* were mainly infected in Germany. Five persons with diphtheria died.

**What are the implications of your findings for public health?**

Diphtheria remains rare in Germany, but since 2023 more people have become infected in the country than previously. To prevent further disease, including severe illness or even death, it is important to raise awareness of diphtheria and better understand who is not protected enough by vaccination.

## Introduction

Diphtheria was long considered a forgotten disease in Germany owing to the substantial decline in cases following the introduction of a diphtheria-toxoid-containing vaccine in 1925 [1]. Since 2001, diphtheria caused by diphtheria-toxin-producing *Corynebacterium (C.) diphtheriae* has been a nationally notifiable disease under the German Infection Protection Act. In 2017, statutory notification was expanded to other toxigenic species including *C. ulcerans.* The toxin-mediated disease presents mainly as respiratory and/or cutaneous disease. In Germany, the Standing Committee on Vaccination (STIKO) recommends primary vaccination at 2, 4 and 11 months of age, with booster doses at 5–6 and 9–16 years, and further boosters every 10 years [2]. In Germany, 89% of 6-year-olds have received the complete primary vaccination series, but coverage for adolescent booster vaccination is only 57% among 15-year-olds [3].

Before 2022, most diphtheria cases in Germany and other European countries were caused by *C. ulcerans* [4-6]. Since then, an outbreak of *C. diphtheriae* infections among newly arriving migrants was recorded in western Europe [7-10]. Subsequently, small secondary outbreaks occurred in Germany; a cutaneous diphtheria outbreak affecting people experiencing homelessness in 2023 and a respiratory diphtheria outbreak in 2024 [11,12]. Other European countries, namely Switzerland and Poland, reported three cases among individuals aged > 60 years and two cases among people experiencing homelessness [13,14]. Notably, these cases had the same sequence type (ST)-574 [15].

Infections with non-toxigenic *tox* gene-bearing (NTTB) strains of *Corynebacterium* spp. occur rarely. These strains may serve as a *tox* gene reservoir but do not cause toxin-mediated diphtheria. The prevalence is uncertain, particularly as they may disproportionately affect people experiencing homelessness and people who use alcohol or drugs [11,15-17].

Toxigenic *C. diphtheriae* and *C. ulcerans* can cause diphtheria but differ in certain aspects: *C. diphtheriae* is mainly transmitted between humans, nearly exclusively isolated from humans and often linked to importation via travel or migration, whereas *C. ulcerans* is zoonotic and often associated with contact with companion or farm animals [4,5,18,19]. Some studies suggest that *C. ulcerans* infections more often affect older adults [4,6,19]. Substantial knowledge gaps persist regarding population groups at risk for *C. diphtheriae* and *C. ulcerans* infections, clinical outcomes and key exposures in high-income countries where diphtheria does not primarily present as classic respiratory diphtheria.

We describe the epidemiology of *C. diphtheriae* and *C. ulcerans* in Germany using statutory surveillance data between 2017 and 2025, complemented with data from an enhanced surveillance project for a subset of diphtheria cases notified between 2017 and 2022, to inform targeted public health measures, improve early detection and treatment, and guide vaccination and prevention strategies.

## Methods

In this population-based study, we included all diphtheria cases fulfilling the Robert Koch Institute’s reference case definition: (i) laboratory-confirmed *Corynebacterium* spp. infection, confirmed either by detection of the *tox* gene with PCR (i.e. *tox* positive) or by detection of toxin production through Elek test (i.e. toxigenic), accompanied by corresponding clinical symptoms, or (ii) clinical symptoms and an epidemiological link to a laboratory-confirmed case [20]. Clinical signs of diphtheria were defined as adherent pseudomembranous lesions, sore throat, nasal discharge, skin or conjunctival lesions, or diphtheria-related death.

Data sources included statutory surveillance (1 January 2017–31 December 2025) and the enhanced diphtheria surveillance project (1 January 2017–31 December 2022).

### Statutory surveillance

Notification of diphtheria cases is mandatory in Germany based on clinical symptoms (clinicians) or detection of toxigenic *Corynebacterium* spp. (laboratories). Submission of clinical samples to the German National Conciliary Laboratory for Diphtheria is recommended, where *tox* positive and toxigenic strains are confirmed using real-time PCR [21], lateral flow immunoassay [22] and Elek test [23].

Clinical manifestations were classified as cutaneous diphtheria, respiratory diphtheria or double manifestation. Respiratory diphtheria with pseudomembranes, i.e. usually thick, often white and adherent exudate in the nose, pharynx, tonsils or larynx, were classified as classic respiratory diphtheria; respiratory symptoms without pseudomembranous lesions were classified as mild diphtheria [24]. We defined double manifestation as cutaneous diphtheria with respiratory colonisation or infection or symptoms consistent with mild or classic respiratory diphtheria.

Likely route of acquisition was defined as travel-associated (travel abroad within 21 days before detection), migration-associated (migration to Germany within 21 days before detection or stay in an asylum reception centre), autochthonous (Germany as country of infection) or unknown. All migration- or travel-associated cases were classified as imported.

Vaccination status was defined as follows: (i) fully vaccinated with ≥ 3 vaccine doses received (primary vaccination series completed); (ii) partially vaccinated with one or two vaccine doses reported; (iii) never vaccinated with no vaccination reported; (iv) unknown with no information to be determined. Vaccinations within 30 days before notification were not considered when determining vaccination status as they likely presented catch-up or post-exposure vaccinations.

Place of notification indicated the district and federal state of notification classified according to the Federal Institute for Research on Building, Urban Affairs and Spatial Development (BBSR) categories of urbanisation [25].

### Enhanced surveillance project

In the enhanced diphtheria surveillance project, additional information on all statutory diphtheria cases was collected to better understand case characteristics and exposures with a focus on *C. ulcerans*. The project was integrated into the statutory surveillance in April 2019 and allowed for the collection of additional information without duplicating case identification efforts.

Local public health authorities collected information retrospectively (cases reported between January 2017 and April 2019) and prospectively (cases reported between May 2019 and December 2022) using a standardised four-page questionnaire. Information was collected from the case and/or their attending physician. Collected information included, among others, contact with companion and farm animals, consumption of unpasteurised milk products, co-infections and wound locations.

While the project initially allowed for more detailed case information on the first imported outbreak cases among newly arriving migrants, the project was ended at the end of 2022 as rising case numbers increased the workload for the public health authorities, and the questionnaire no longer added relevant information, e.g. migration route.

### Statistical analysis

Descriptive analyses were performed to examine clinical, demographic and epidemiological characteristics. Population estimates from the Federal Statistics Office were used to calculate incidence rates [26]. We compared case characteristics (age, sex, place of notification and hospitalisation) using chi-square and Fisher’s exact tests to identify differences between species, and autochthonous and imported *C. diphtheriae* cases. To describe changes over time, we defined different epidemiological phases and calculated incidence rate ratios (IRRs) by phase. To quantify trends in autochthonous cases, we fitted a Poisson regression model to the case time series. Time was modelled in calendar quarters, and IRRs were estimated per quarter. A significance level of 0.05 was used for all analyses. Statutory surveillance data were derived using the SurvNet@RKI software, as of 28 February 2026 (https://survnet.rki.de/). Data were analysed using R version 4.3.0 (https://www.r-project.org/about.html).

## Results

A total of 512 diphtheria cases were reported between 2017 and 2025 in Germany, a country with approximately 83 million inhabitants. *Corynebacterium diphtheriae* accounted for 68% of cases (346/512; average annual incidence: 0.045/100,000 population) and *C. ulcerans* for 32% of cases (166/512; average annual incidence: 0.022/100,000 population).

### Microbiological results

Overall, 79% (405/512) of diphtheria infections with a toxigenic strain were confirmed using Elek test (Table 1). Seven epidemiologically linked cases were reported in 2023, and for the remaining 100 cases, only the presence of *tox* gene was reported. While confirmation of toxigenicity by Elek test is recommended, though not mandatory in Germany, the proportion of Elek-confirmed cases increased during the study period from 23 of 51 cases in 2017–2019 to 74 of 103 (72%) cases in 2024–2025.

**Table 1:** Characteristics of diphtheria cases with *Corynebacterium diphtheriae* and *Corynebacterium ulcerans*, by epidemiological phase, Germany, 2017–2025 (n = 512)^a^

Characteristic	*Corynebacterium diphtheriae* (n = 346)						*Corynebacterium ulcerans* (n = 166)					
	Total		2017–2019 (n = 12)	2020–2021 (n = 19)	2022–2023 (n = 261)	2024–2025 (n = 54)	Total		2017–2019 (n = 39)	2020–2021 (n = 27)	2022–2023 (n = 49)	2024–2025 (n = 51)
	n	%					n	%				
Average yearly number of cases	38		4	10	130	27	18		13	14	24	26
Average yearly incidence (per 100,000)	0.045		0.005	0.012	0.154	0.032	0.022		0.016	0.017	0.028	0.031
Case classification												
Laboratory-confirmed toxigenic	280	80.9	6	8	233	33	125	75.3	17	18	41	49
Laboratory-confirmed *tox* gene	59	17.1	6	11	21	21	41	24.7	22	9	8	2
Epidemiologically linked	7	2.0	0	0	7	0	0		0	0	0	0
Clinical manifestation^b^												
Classic respiratory	7	2.0	0	1	4	2	2	1.2	1	0	0	1
Mild respiratory	15	4.3	0	0	11	4	6	3.6	1	1	2	2
Cutaneous	306	88.4	12	18	232	44	154	92.8	37	26	43	48
Double manifestation	18	5.2	0	0	14	4	4	2.4	0	0	4	0
Sex (n = 510)^c^												
Male	314	90.8	9	18	244	43	108	65.1	30	20	27	31
Female	30	8.6	3	1	15	11	58	34.9	9	7	22	20
Age group (years) (n = 508)^d^												
< 15	39	11.3	2	4	32	1	2	1.2	0	1	1	0
15–29	222	64.2	3	10	200	9	5	3.0	1	0	3	1
30–44	37	10.7	1	2	19	15	9	5.4	2	0	2	5
45–64	34	9.8	4	2	7	21	59	35.5	15	10	15	19
≥ 65	12	3.5	1	1	3	7	89	53.6	20	16	28	25
Place of notification^e^												
**Rural**	**85**	**24.6**	**1**	**8**	**69**	**7**	**78**	**47.0**	**17**	**18**	**18**	**25**
Sparsely populated	37	10.7	1	3	29	4	41	24.7	9	12	10	10
Densely populated	48	13.9	0	5	40	3	37	22.3	8	6	8	15
**Urban**	**261**	**75.4**	**11**	**11**	**192**	**47**	**88**	**53.0**	**22**	**9**	**31**	**26**
Urban district	78	22.5	2	2	67	7	52	31.3	13	4	20	15
Small city	105	30.3	4	4	85	12	19	11.4	5	4	6	4
Large city	78	22.5	5	5	40	28	17	10.2	4	1	5	7

^a^ Case definition: any case fulfilling the case definition of Robert Koch Institute [20]: a person with a laboratory-confirmed toxigenic or *tox* positive isolate of *Corynebacterium (C.) diphtheriae* or *C. ulcerans* or a case with an epidemiological link to a laboratory confirmed diphtheria case and relevant clinical symptoms.

^b^ Classic respiratory manifestation: any case with pseudomembranes in the nose, pharynx, tonsils or larynx, all other cases with respiratory manifestations are considered as mild.

^c^ Sex of two cases with *C. diphtheriae* in 2022–2023 was reported as unknown.

^d^ Age was unknown for two *C. diphtheriae* cases and two *C. ulcerans* cases, with one case of each species reported in 2018 and one in 2025.

^e^ Reported residence of each case at the time of notification was based on the Federal Institute for Research on Building, Urban Affairs and Spatial Development (BBSR) categories, distinguishing sparsely populated rural districts, more densely populated rural districts, urban districts (< 100,000 inhabitants), small cities (100,000–500,000 inhabitants) and large cities (> 500,000 inhabitants).

### Epidemiological phases

Before 2022, *C. diphtheriae* was less frequently reported than *C. ulcerans* with 4 vs 13 (2017–2019) and 10 vs 14 (2020–2021) cases, respectively. We identified four epidemiological phases: pre-emergence (2017–2019), early re-emergence (2020–2021), outbreak (2022**–**2023) and re-emergence (2024–2025) (Figure 1). Case characteristics by year are available in Supplementary Table S1. The outbreak phase (2022–2023) was primarily associated with the European-wide outbreak of *C. diphtheriae* among newly arriving migrants with an average of 130 annual *C. diphtheriae* cases compared with 24 *C. ulcerans* cases. In the re-emergence phase (2024–2025), both species were equally reported (27 vs 26 average annual cases). The IRRs were consistent with these patterns: compared with the pre-emergence phase, *C. diphtheriae* incidence increased sharply during the outbreak phase (IRR = 32.1) and remained elevated in the re-emergence phase (IRR = 6.71).

**Figure 1:**
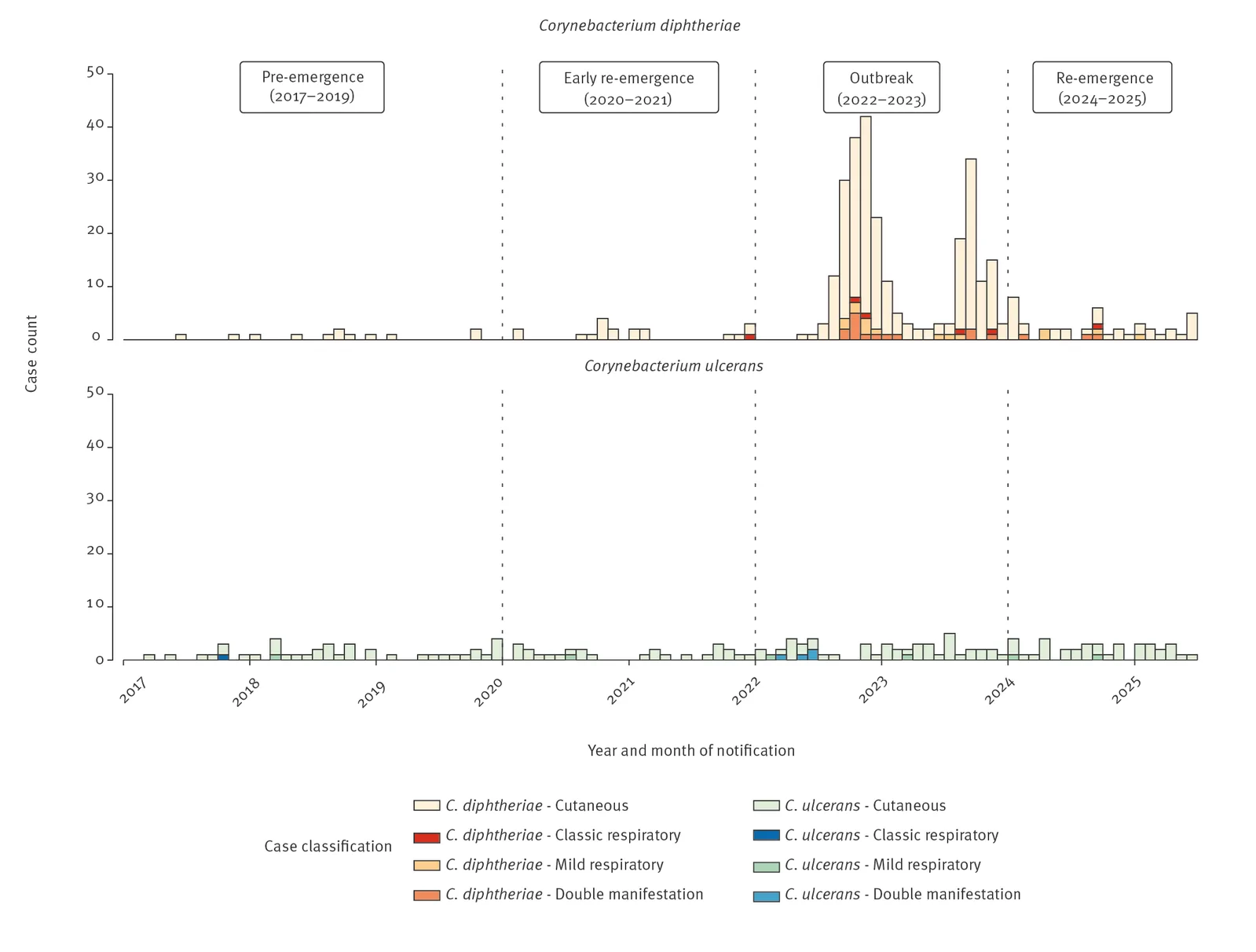
*Corynebacterium diphtheriae* and *Corynebacterium ulcerans* cases, by year and month of notification, clinical manifestation and epidemiological phase, Germany, 2017–2025 (n = 512)

Infections were predominantly cutaneous (460/512; 90%) (Table 1). Double manifestations (22/512; 4%) were as common as mild respiratory manifestations (21/512; 4%). Among *C. diphtheriae* cases, only one respiratory infection was reported before 2022 among the 31 cases. In the outbreak phase (2022–2023), 6% of cases (15/261) had a respiratory disease and 5% (14/261) had double manifestations. In the re-emergence phase, six respiratory and four double manifestations were recorded among 54 cases. Cases with *C. ulcerans* were mostly cutaneous with sporadic respiratory manifestations observed throughout the study period.

### Likely route of acquisition

The likely route of acquisition of *C. diphtheriae* shifted across the epidemiological phases (Table 2). During the pre-emergence phase (2017–2019), all 12 *C. diphtheriae* infections were travel-associated. In the early re-emergence (2020–2021) and outbreak (2022–2023) phases, migration-associated infections predominated, accounting for 14 of 16 cases and 232 of 256 (89%) cases with available information, respectively. In the re-emergence phase (2024–2025), most cases were autochthonous (35/46). In contrast, all 145 *C. ulcerans* cases with available information were autochthonous.

**Table 2:** Exposures and vaccination status among diphtheria cases, by species and epidemiological phase, Germany, 2017–2025 (n = 512)^a^

Characteristic	*Corynebacterium diphtheriae* (n = 346)						*Corynebacterium ulcerans* (n = 166)					
	Total		2017–2019 (n = 12)	2020–2021 (n = 19)	2022–2023 (n = 261)	2024–2025 (n = 54)	Total		2017–2019 (n = 39)	2020–2021 (n = 27)	2022–2023 (n = 49)	2024–2025 (n = 51)
	n	%					n	%				
Likely route of transmission^b^												
Cases with information	330	95.4	12	16	256	46	145	87.3	39	27	38	41
Autochthonous	45	13.6	0	1	9	35	145	87.3	39	27	38	41
Travel-associated	33	10.0	12	1	15	5	0	0	0	0	0	0
Migration-associated	252	76.4	0	14	232	6	0	0	0	0	0	0
Unknown	16	4.6	0	3	5	8	21	12.7	0	0	11	10
Comorbidities												
Cases with information	75	21.7	2	7	64	2	46	27.7	13	7	19	7
At least one	7	9.3	1	1	4	1	33	71.7	10	6	12	5
None	68	90.7	1	6	60	1	13	28.3	3	1	7	2
Unknown	271	78.3	10	12	197	52	120	72.3	26	20	30	44
Vaccination status^c^												
Cases with information	133	38.4	10	9	99	15	112	67.5	27	17	33	35
Fully vaccinated	2	1.5	1	1	0	0	3	2.7	1	0	2	0
Partially vaccinated	70	52.6	8	3	51	8	83	74.1	17	13	26	27
Never vaccinated	61	45.9	1	5	48	7	26	23.2	9	4	5	8
Unknown	213	61.6	2	10	162	39	54	32.5	12	10	16	16
Contact with companion animal												
Cases with information	42	12.1	5	6	30	1	89	53.6	24	13	32	20
Reported	4		2	0	2	0	70	78.7	17	8	25	20
Not reported	38		3	6	28	1	19	21.3	7	5	7	0
Unknown	304	87.9	7	13	231	53	77	46.4	15	14	17	31
Farm animal contact												
Cases with information	31	9.0	2	4	25	0	30	18.1	8	6	16	0
Reported	0		0	0	0	0	3-		1	0	2	0
Not reported	31		2	4	25	0	27		7	6	14	0
Unknown	315	91.0	10	15	236	54	136	81.9	31	21	33	51
Consumption of raw unpasteurised milk products												
Cases with information	7	2	1	0	6	0	24	14.5	5	5	14	0
Reported	0		0	0	0	0	2		0	0	2	0
Not reported	7		1	0	6	0	22		5	5	12	0
Unknown	339	98	11	19	255	54	142	85.5	34	22	35	51

^a^ Percentages are calculated using the number of cases with available information as the denominator. No percentages are indicated if the denominator is < 45 cases.

^b^ Autochthonous: case had stayed in Germany 21 days before detection; migration-associated: case had migrated to Germany within 21 days before detection or stayed at the time of notification in an asylum reception centre; travel-associated: case was travelling abroad 21 days before detection.

^c^ Fully vaccinated: ≥ 3 vaccine doses received (primary vaccination series completed); partially vaccinated: 1 or 2 vaccine doses. Vaccinations included if vaccinated 30 days before disease onset or, if not available, date of notification.

### Demographics, vaccination and exposure history

Diphtheria cases were predominantly males, with a higher predominance among *C. diphtheriae* cases compared with *C. ulcerans* cases (91% vs 65%; chi-square test: p < 0.001) (Table 1). Cases with *C. diphtheriae* were young, with a median age of 21 years (quartile (Q)1–Q3: 17–29 years) and 64% (222/346) were aged 15–29 years. This was especially pronounced during the outbreak phase (2022–2023) when 77% (200/261) of cases were adolescents or young adults. Cases with *C. ulcerans* were a median age of 67 years (Q1–Q3: 57–76 years). Among those with information available on comorbidities, few *C. diphtheriae* cases (9%, 7/75) had comorbidities whereas it was more common among *C. ulcerans* cases (33/46) (Table 2).

Information on vaccination status was available for 38% (133/346) of *C. diphtheriae* and 67% (112/166) of *C. ulcerans* cases. Among these, only two of 133 (2%) *C. diphtheriae* and three of 112 (3%) *C. ulcerans* cases were fully vaccinated (≥ 3 doses). More than half of the *C. diphtheriae* and *C. ulcerans* cases were partially vaccinated (*C. diphtheriae*: 53%; 70/133 and *C. ulcerans*: 74%; 83/112).

Contact with companion animals was common among *C. ulcerans* cases (70/89; 79%), where information was available. For cases with *C. diphtheriae*, information was rarely available but none of those reported farm animal contact or consumption of raw unpasteurised milk products. For cases with *C. ulcerans,* three of 30 cases reported farm animal contact and two of 24 reported consumption of raw unpasteurised milk products.

### Geographical distribution

Cases with *C. diphtheriae* and *C. ulcerans* were reported throughout Germany (Figure 2). A geographical concentration of *C. diphtheriae* cases was observed during the outbreak phase (2022–2023) in south-western and north-eastern Germany and during the re-emergence phase (2024–2025) in Frankfurt am Main and Berlin. Notably, the municipality of Freiburg im Breisgau in south-western Germany accounted for 50 of 261 cases (19%) in the outbreak phase. No distinct geographical pattern was observed for *C. ulcerans* cases. Incidence rates per 100,000 inhabitants indicated no geographical concentration. Incidence maps by species and epidemiological phase can be found in Supplementary Figure S1. While *C. diphtheriae* cases were primarily notified in urban districts (261/346; 75%), *C. ulcerans* cases were from both urban and rural districts (Table 1).

**Figure 2:**
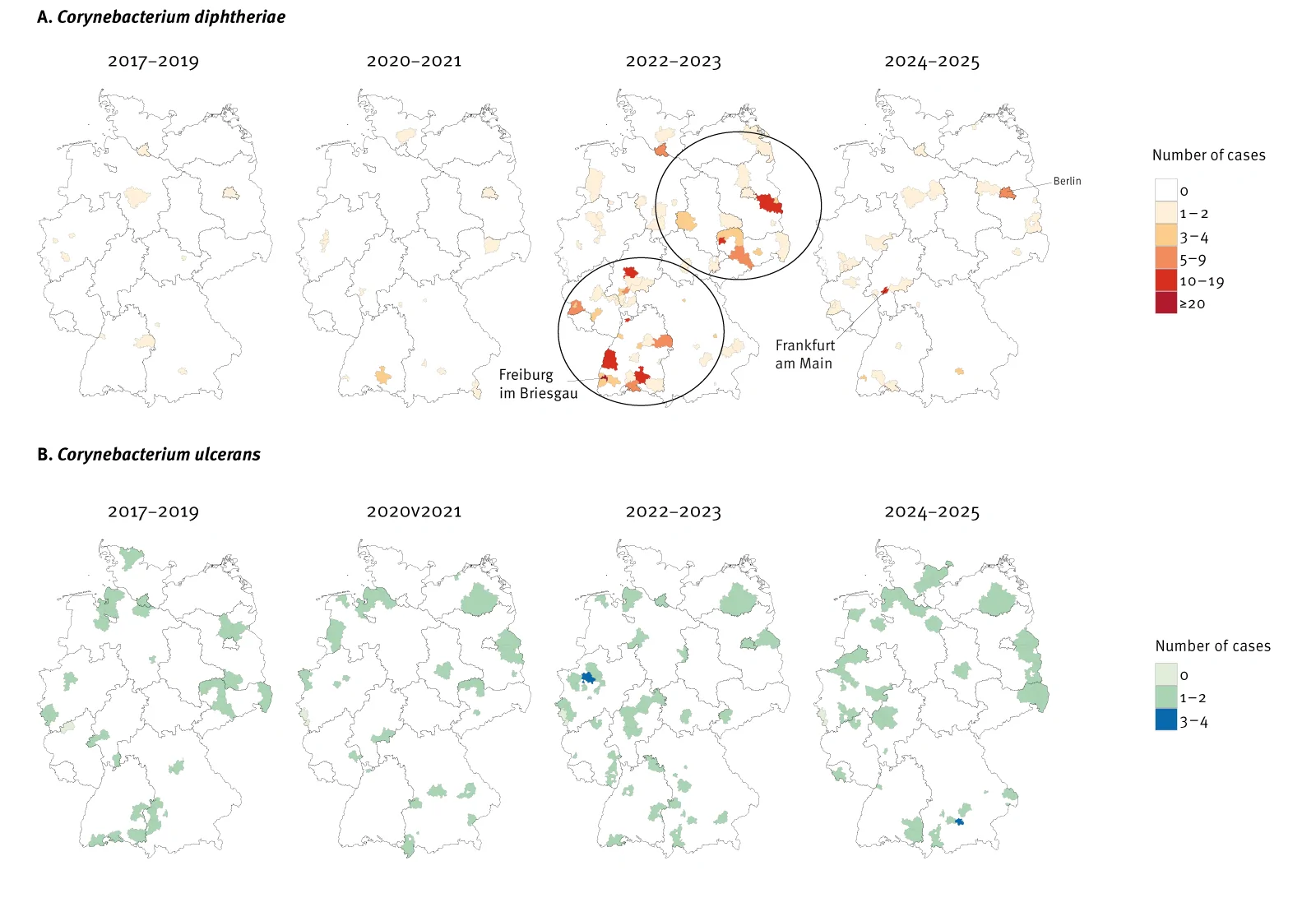
Geographical distribution of cases with *Corynebacterium diphtheriae* (Panel A) and *Corynebacterium ulcerans* (Panel B), by district and epidemiological phase, Germany, 2017–2025 (n = 512)

### Hospitalisation, length of hospitalisation and deaths

No differences in hospitalisation status were observed between *C. diphtheriae* and *C. ulcerans* cases. We present hospitalisation, length of hospitalisation and deaths among diphtheria cases by species in Supplementary Table S2. Cases with any respiratory symptom were more commonly hospitalised than cutaneous cases (24/52 vs 110/417; chi-square test: p < 0.01) (Table 3). Median length of hospitalisation across all disease manifestations was 7 days (Q1–Q3: 3–10 days). During the study period, 10 cases died: five deaths were attributed to diphtheria (case fatality rate: 1%) (Table 3). Three respiratory cases passed away, all with *C. diphtheriae* ST-574: in early 2024, a healthcare worker in their seventies; in late 2024, a care home resident in their eighties, both with an unknown vaccination status; and in early 2025, an unvaccinated school-aged child. Two cutaneous cases, both with *C. ulcerans*, passed away: in late 2021, a person in their eighties with an unknown vaccination status; and in mid-2023, a previously vaccinated person in their fifties with unknown number of doses who developed sepsis, also confirmed by toxigenic *C. ulcerans* blood culture isolate.

**Table 3:** Hospitalisation, length of hospitalisation and deaths among diphtheria cases, by clinical manifestation, Germany, 2017–2025 (n = 512)

Characteristic	Total		Classic respiratory (n = 9)	Mild respiratory (n = 21)	Cutaneous (n = 460)	Double manifestation (n = 22)
	n	%				
Cases with hospitalisation data	476	93	9	21	417	22
Hospitalised cases	136	28.6	5	9	110	10
Hospitalised cases with known length of hospitalisation	111	81.6	2	5	87	6
Median length of hospitalisation in days (Q1–Q3)	7 (3–10)		6 (4–8)	7 (4–10)	2 (0–15)	2 (1–4)
Deaths	10		1	2	7	0
Deaths due to diphtheria	5		1	2	2	0
Deaths due to other cause	5		0	0	5	0

### Autochthonous cases

Over the study period, the number of autochthonous *C. diphtheriae* cases increased (IRR per quarter = 1.19; 95% confidence interval (CI): 1.14–1.27; p < 0.001), while the number of autochthonous *C. ulcerans* cases remained stable (IRR per quarter = 1.02, 95% CI: 1–1; p < 0.05). Compared with imported cases, most autochthonous *C. diphtheriae* cases were aged ≥ 45 years (23/45) (Table 4). Also, a higher proportion of autochthonous cases were females (autochthonous: 12/45 vs imported: 16/283; chi-square test: p < 0.001), manifestations with respiratory involvement were more frequent (10/45 vs 30/283; Fisher’s exact test: p < 0.05) and hospitalisations were more common (36/45 vs 83/283; Fisher’s exact test: p < 0.001).

**Table 4:** Characteristics of autochthonous and imported cases with *Corynebacterium diphtheriae*, Germany, 2017–2025 (n = 328)

Characteristic	Autochthonous (n = 45)		Imported (n = 283)^a^		p value^b^
	n	%	n	%	
Age group (years)					
< 15	1	2.2	37	13.1	< 0.001
15–29	4	8.9	208	73.5	
30–44	16	35.6	18	6.4	
45–64	18	40.0	14	4.9	
≥ 65	5	11.1	5	1.8	
Sex^c^					
Male	33	73.3	267	94.3	< 0.001
Female	12	26.7	16	5.7	
Place of notification					
Rural	6	13.3	75	26.5	0.057
Urban	39	86.7	208	73.5	
Clinical manifestation					
Classic respiratory diphtheria	2	4.4	5	1.8	0.048
Mild respiratory diphtheria	5	11.1	10	3.5	
Cutaneous diphtheria	35	77.8	253	89.4	
Double manifestation	3	6.7	15	5.3	
Hospitalisation status					
Hospitalised	36	80.0	83	29.3	< 0.001
Not hospitalised	8	17.8	171	60.4	
Unknown	1	2.2	29	10.2	

^a^ Autochthonous: case had stayed in Germany 21 days before detection; migration-associated: case had migrated to Germany within 21 days before detection or stayed at the time of notification in an asylum reception centre; travel-associated: case was travelling abroad 21 days before detection.

^b^ For two categories, chi-square test was used, for > 2 categories, Fisher’s exact test.

^c^ Two cases with unknown sex were excluded.

### Co-infections and wound location in cutaneous diphtheria cases

The enhanced diphtheria surveillance project provided additional information for a subset of 111/268 (41%) of diphtheria cases and for 101/241 (42%) of cutaneous diphtheria cases notified between 2017 and 2022. Information on co-infections was available for 94 of 101 (93%) cases. Among those, 77 (82%) reported co-infections (*C. diphtheriae* cases: 58/67; 87% and *C. ulcerans* cases: 19/27). We present the number of co-infections in more detail in Supplementary Table S3. Among the *C. diphtheriae* cases, *S. pyogenes*, *S. aureus* and meticillin-resistant *S. aureus* (MRSA) were most often reported, whereas *S. aureus*, *P. mirabilis* and *E. faecalis* predominated among the *C. ulcerans* cases. We present a list of all detected co-infection agents in cutaneous diphtheria cases in Supplementary Table S4. For 25 of 28 *C. diphtheriae* cases and 16 of 17 *C. ulcerans* cases with information, wounds were most often located on legs or on feet. For *C. diphtheriae* cases, lower legs were most often affected (17/28), whereas for *C. ulcerans* cases wounds were most common on the feet (11/17). We include further details on wound locations in cutaneous cases by species in Supplementary Table S5.

## Discussion

Between 2017 and 2025, the epidemiology of diphtheria in Germany changed substantially leading to its re-emergence. Although diphtheria remains rare, *C. diphtheriae* infections shifted from a phase of predominantly travel- and migration-associated cases to increasingly autochthonous acquisition, with a 19% increase of autochthonous cases per quarter, since 2024. Autochthonous cases were more likely to present with more severe respiratory manifestations suggesting possible underdetection of milder infections. The occurrence of severe respiratory forms, hospitalisations and deaths highlights the renewed clinical and public health relevance of diphtheria in Germany. In contrast, *C. ulcerans* infections were consistently autochthonous and predominantly affected older individuals who frequently reported contact with companion animals.

Following the European-wide *C. diphtheriae* outbreak in 2022, Germany saw a second peak of cases in autumn/winter 2023. Other affected countries by the European-wide outbreak, such as France and the Netherlands, did not report a continued increase in cases [27]. Although Switzerland reported three autochthonous cases, two in older adults and one in a person experiencing homelessness [13], no other European country than Germany has reported a similar sustained shift in acquisition pattern. The demographic profile of the cases also shifted. While the outbreak phase mainly involved young newly arriving male migrants, autochthonous cases were primarily middle-aged and older individuals and people experiencing homelessness or people using alcohol or drugs [11,15]. As of July 2025, molecular analyses have linked 21 autochthonous cases to the same ST-574, suggesting sustained local transmission [15].

Several factors may have contributed to the increased detection. The *C. diphtheriae* outbreak in 2022 increased awareness among clinicians and public health authorities. For example, a concentration of *C. diphtheriae* cases in Freiburg im Breisgau likely reflects the presence of a large asylum reception centre in the municipality [7]. Active case finding during an outbreak investigation in Frankfurt am Main in 2023 may have improved detection among people experiencing homelessness [11]. Still, together with evidence from reported outbreaks with documented or suspected local transmission [11,12,15], the shift in acquisition pattern over time, increasing trend in autochthonous cases and newly affected population groups indicate a true re-emergence.

Although the recent re-emergence of diphtheria in Germany was primarily driven by *C. diphtheriae*, our findings show that both *C. diphtheriae* and *C. ulcerans* can cause severe disease leading to death. This is consistent with previous studies documenting that infections caused by toxigenic *C. ulcerans* can be clinically indistinguishable from those caused by toxigenic *C. diphtheriae* [4,28,29]. Respiratory diphtheria is historically considered the most severe form, though our findings highlight that rare, severe and even fatal outcomes of cutaneous diphtheria can also occur. While hospitalisation was common, it may reflect precautionary management (e.g. specialist referral, isolation measures) rather than disease severity alone. Frequency and duration of hospitalisation were comparable between the two species, but the populations affected differed. Infections with *C. ulcerans* primarily affected individuals aged over 65 years which frequently report comorbidities possibly contributing to frequent hospitalisation.

Reported cases likely present only the tip of the iceberg of diphtheria infections. Most infections in the study were cutaneous, which is in line with studies from other high-income countries [4,5]. Cutaneous diphtheria often presents with non-specific symptoms and may therefore remain undetected. Undiagnosed cutaneous infections may possibly last for several months and, if caused by *C. diphtheriae*, contribute to ongoing circulation and transmission thus making improved detection essential for understanding transmission dynamics [12,30]. Among the small sample of cutaneous cases with available information, co-infections were common, which is in line with other studies [31]. As non-toxigenic corynebacteria are commonly present in normal skin flora, potentially toxigenic *Corynebacterium* spp. may be overlooked during routine microbiological examination of wound samples, particularly in polymicrobial infections [32]. Therefore, in non-healing wounds, selection for species level identification of *Corynebacterium* spp. should be considered even when other pathogens such as *S. pyogenes* and *S. aureus* are identified in wound isolates.

Simultaneous cutaneous and respiratory involvement may contribute to transmission dynamics potentially facilitating switching between cutaneous and respiratory manifestation [12]. While double manifestations were described before, it is unclear how often they occur [4,7]. Although this study does not allow for the distinction between asymptomatic respiratory carriage and respiratory symptoms occurring alongside cutaneous disease, double manifestations were nearly as common as mild respiratory manifestations. Examination of cutaneous cases for respiratory carriage and respiratory cases for skin lesions is thus essential. Since June 2025, double manifestations have been incorporated in the German electronic notification software SurvNet and are now systematically recorded.

Among *C. ulcerans* cases, pet ownership and comorbidities were frequently reported. This reaffirms the understanding of *C. ulcerans* infections as primarily zoonotic and highlights the role of companion animals. A case–case study suggested that the risk of infection may be higher among individuals with diabetes mellitus [19]. Collecting key exposures in statutory surveillance, including contact with companion animals, could continue to improve our understanding of risk factors. Recording companion animal contact may also facilitate collaboration with veterinary services. If toxigenic *C. ulcerans* is confirmed in a companion animal, the United Kingdom Health Security Agency recommends antimicrobial treatment of the animal and all other animals in the same household, when appropriate, thereby helping to prevent (re)infection [33].

Vaccination is the most effective protection against severe forms of diphtheria. Diphtheria vaccines are based on the *C. diphtheriae* toxoid, and although cross-protection is generally assumed, the extent of protection for *C. ulcerans* remains unclear [28]. Germany lacks an electronic immunisation registry. Vaccination status was unavailable for many cases in this study, possibly due to challenges in case investigations such as recalling childhood vaccinations, missing documentation among newly arriving migrants and/or language barriers. The low number of cases who had received at least three doses and thus completed the primary vaccination series did not indicate vaccine failure. Due to the high incompleteness, no further inference is possible.

While vaccination coverage with completed primary vaccination series among children is generally high, immunity among middle-aged and older people in Germany is unclear with the latest seroprevalence study conducted in 1996 [34]. However, data from other European countries suggest varying but largely insufficient protection among middle-aged people and an increasing proportion of seronegative individuals with age [35,36]. Given that adults aged over 60 years are affected by both autochthonous *C. diphtheriae* and *C. ulcerans* infections, booster vaccinations in older age groups should be emphasised to address waning immunity [34]. Newly arriving migrants and individuals with unknown vaccination status should receive catch-up vaccinations to complete primary vaccination series and receive recommended boosters as soon as possible. Serological studies are needed to assess population-based immunity and immunity especially among older adults and people experiencing homelessness and identify appropriate vaccination strategies to reach undervaccinated people.

The following limitations should be taken into consideration. Firstly, mild respiratory or cutaneous infections may remain undetected. To address potential underdetection, we interpreted trends over time in light of changes in awareness and case detection. Secondly, the classification of likely route of acquisition relied on available information on travel and migration history. Misclassifications may have occurred due to challenges in obtaining metadata, particularly among mobile populations such as newly arriving migrants or people experiencing homelessness. We interpreted trends in autochthonous cases together with other epidemiological indicators such as changes in affected populations. Thirdly, data on the key variables were often missing, e.g. vaccination status and consumption of raw unpasteurised milk products. Information from the enhanced diphtheria surveillance project was only available for a non-representative subset of cases with migration-associated cases being over-represented compared with all reported diphtheria cases [37]; this may limit the generalisability. We transparently reported the number of cases with missing information. Fourthly, some subgroup analyses, including deaths, were based on small numbers and should therefore be interpreted with caution. Finally, differences in case definitions and diagnostic practices may affect comparability with other surveillance systems. For example, RKI’s reference case definitions exclude asymptomatic laboratory-confirmed cases but includes cases with detection of the *tox* gene but without toxin production demonstrated via Elek-Test, which cannot cause toxin-mediated disease.

## Conclusion

Diphtheria is re-emerging in Germany. While this study provides a better understanding of affected population groups, there is a need to further investigate immunisation gaps to identify undervaccinated people. In the meantime, strengthening routine vaccination will be key to prevent severe cases and deaths, particularly catch-up vaccinations for those with unknown or incomplete vaccination status and booster doses for older individuals. Given its renewed relevance, clinicians, microbiologists and public health services should re-familiarise themselves with diphtheria to ensure prompt diagnosis, treatment and prevention of further spread.

## Data Availability

The data from mandatory notification system have limited and restricted access. Further detailed information on outbreak data is protected under German law.

## Supplementary Data

895000 Supplementary Material
