# Supplementary material for: Re-emergence of diphtheria in Germany: a national population-based surveillance study of Corynebacterium diphtheriae and Corynebacterium ulcerans, 2017 to 2025
Source: Euro Surveill. 2026 Nov 12;31(45):2600348. doi: 10.2807/1560-7917.ES.2026.31.45.2600348 (PMC13768766; doi:10.2807/1560-7917.ES.2026.31.45.2600348)
Supplement: Supplementary file 1 [file 26-00348_SCHNEIDER_Supplement.pdf]

Supplement

Title: Re-emergence of diphtheria in Germany: a national population-based surveillance study of *C. diphtheriae* and *C. ulcerans*, 2017–2025

Authors: L.E. Schneider, F. Badenschier, K. Bengs, M. Martín-Sánchez, C. Wagner-Wiening, Külper-Schiek, W., O. Wichmann, A. Berger, A. Sing, C. Rau, D. Perriat

This supplementary material is hosted by Eurosurveillance as supporting information alongside the article „Re-emergence of diphtheria in Germany: a national population-based surveillance study of *C. diphtheriae* and *C. ulcerans*, 2017–2025“, on behalf of the authors, who remain responsible for the accuracy and appropriateness of the content. The same standards for ethics, copyright, attributions and permissions as for the article apply. Supplements are not edited by *Eurosurveillance* and the journal is not responsible for the maintenance of any links or email addresses provided therein.

Supplementary Table S1: Selected case characteristics of *C. diphtheriae* and *C. ulcerans* cases by year, Germany, 2017 – 2025 (n=512)

	C. diphtheriae		*C. ulcerans*		*C. diphtheriae*									*C. ulcerans*								
	Total	%	Total	%	2017	2018	2019	2020	2021	2022	2023	2024	2025	2017	2018	2019	2020	2021	2022	2023	2024	2025
**Total cases (n)[a]**	346		166		2	7	3	10	9	150	111	29	25	8	19	12	14	13	22	27	24	27
**Case classification n**																						
Laboratory-confirmed toxigenic (positive Elek test)	280	80.9	125	75.3	0	5	1	3	5	140	93	23	10	2	9	6	7	11	19	22	22	27
Laboratory-confirmed *tox*-positive (positive qPCR for *tox*-gene)	59	17.1	41	24.7	2	2	2	7	4	10	11	6	15	6	10	6	7	2	3	5	2	0
Epidemiologically linked (Epi-link to laboratory-confirmed case)	7	2	0	-	0	0	0	0	0	0	7	0	0	0	0	0	0	0	0	0	0	0
**Clinical manifestation n[b]**																						
Classic respiratory	7	2	2	1.2	0	0	0	0	1	2	2	1	1	1	0	0	0	0	0	0	0	1
Mild respiratory	15	4.3	6	3.6	0	0	0	0	0	8	3	3	1	0	1	0	1	0	1	1	2	0
Cutaneous	306	88.4	154	92.8	2	7	3	10	8	131	101	22	22	7	18	12	13	13	17	26	22	26
Double manifestation	18	5.2	4	2.4	0	0	0	0	0	9	5	3	1	0	0	0	0	0	4	0	0	0
**Sex n**																						
Male[c]	314	90.8	108	65.1	2	5	2	9	9	150	94	24	19	7	14	9	10	10	13	14	13	18
Female	30	8.7	58	34.9	0	2	1	1	0	0	15	5	6	1	5	3	4	3	9	13	11	9
**Age (in years)[d]**																						
Age Group n																						
Age <15	39	11.3	2	1.2	0	2	0	3	1	22	10	1	0	0	0	0	1	0	1	0	0	0

	C. diphtheriae		*C. ulcerans*		*C. diphtheriae*									*C. ulcerans*								
	Total	%	Total	%	2017	2018	2019	2020	2021	2022	2023	2024	2025	2017	2018	2019	2020	2021	2022	2023	2024	2025
Age 15–29	222	64.2	5	3	0	2	1	5	5	116	84	7	2	0	1	0	0	0	1	2	0	1
Age 30–44	37	10.7	9	5.4	0	0	1	0	2	11	8	7	8	0	1	1	0	0	1	1	1	4
Age 45–64	34	9.8	59	35.5	1	2	1	1	1	1	6	12	9	5	6	4	6	4	5	10	6	13
Age 65+	12	3.5	89	53.6	1	0	0	1	0	0	3	2	5	3	10	7	7	9	14	14	17	8
**Place of notification n[e]**																						
Rural	85	24.6	78	47	0	1	0	5	3	33	36	7	0	4	7	6	11	7	10	8	13	12
Sparsely Populated Rural District	37	10.7	41	24.7	0	1	0	1	2	11	18	4	0	3	4	2	7	5	7	3	7	3
More densely Populated Rural District	48	13.9	37	22.3	0	0	0	4	1	22	18	3	0	1	3	4	4	2	3	5	6	9
Urban	261	75.4	88	53	2	6	3	5	6	117	75	22	25	4	12	6	3	6	12	19	11	15
Urban District	78	22.5	52	31.3	0	2	0	1	1	45	22	4	3	1	7	5	2	2	8	12	6	9
Small City (100k–500k inhabitants)	105	30.3	19	11.4	1	1	2	2	2	49	36	8	4	2	2	1	1	3	2	4	2	2
Large City (>500k inhabitants)	78	22.5	17	10.2	1	3	1	2	3	23	17	10	18	1	3	0	0	1	2	3	3	4
**Likely route of acquisition n[f]**																						
Autochthonous	45	95.4	145	87.3	0	0	0	0	1	0	9	17	18	8	19	12	14	13	22	16	18	23
Travel-associated	33	13.6	0		2	7	3	0	1	2	13	3	2	0	0	0	0	0	0	0	0	0
Migration-associated	252	9.5	0		0	0	0	7	7	147	85	5	1	0	0	0	0	0	0	0	0	0
Unknown	16	76.4	21	87.3	0	0	0	3	0	1	4	4	4	0	0	0	0	0	0	11	6	4
**Comorbidities n**																						
At least one	7	9.3	33	71.7	0	0	1	0	1	4	0	0	1	3	3	4	2	4	9	3	3	2
None	68	90.7	13	28.3	0	1	0	1	5	59	1	0	1	1	0	2	0	1	7	0	1	1
Unknown	271	78.3	120	72.3	2	6	2	9	3	87	110	29	23	4	16	6	12	8	6	24	20	24
**Vaccination status n[g]**																						
Cases with available information on vaccination[g]	133	38.4	112	73.5	2	5	3	4	5	49	50	11	4	6	11	10	9	8	16	17	14	21

	C. diphtheriae		*C. ulcerans*		*C. diphtheriae*									*C. ulcerans*								
	Total	%	Total	%	2017	2018	2019	2020	2021	2022	2023	2024	2025	2017	2018	2019	2020	2021	2022	2023	2024	2025
Fully vaccinated	2	1.5	3	1.8	0	0	1	0	1	0	0	0	0	1	0	0	0	0	2	0	0	0
Partially vaccinated	70	52.6	83	50	2	5	1	1	2	27	24	5	3	4	8	5	6	7	12	14	13	14
Never vaccinated	61	45.9	26	15.7	0	0	1	3	2	22	26	6	1	1	3	5	3	1	2	3	1	7
**Companion animal contact n**																						
	4	1.3	70	78.7	1	0	1	0	0	2	0	0	0	3	9	5	3	5	18	7	11	9
Yes	38	12.5	19	21.3	0	2	1	1	5	27	1	0	1	1	3	3	2	3	4	3	0	0
No	304	87.9	77	46.4	1	5	1	9	4	121	110	29	24	4	7	4	9	5	0	17	13	18
**Farm animal contact n**																						
Cases with information	31	9	30	18.1	0	1	1	0	4	25	0	0	0	2	1	5	2	4	16	0	0	0
Reported	0	-	3	-	0	0	0	0	0	0	0	0	0	1	0	0	0	0	2	0	0	0
Not reported	31	-	27	-	0	1	1	0	4	25	0	0	0	1	1	5	2	4	14	0	0	0
Unknown	315	91	136	81.9	2	6	2	10	5	125	111	29	25	6	18	7	12	9	6	27	24	27
**Consumption of raw unpasteurized milk products n**																						
Cases with information	7	2	24	14.5	0	0	1	0	0	6	0	0	0	0	1	4	2	3	14	0	0	0
Reported	0	-	2	-	0	0	0	0	0	0	0	0	0	0	0	0	0	0	2	0	0	0
Not reported	7	-	22	-	0	0	1	0	0	6	0	0	0	0	1	4	2	3	12	0	0	0
Unknown	339	98	142	85.5	2	7	2	10	9	144	111	29	25	8	18	8	12	10	8	27	24	27

[a] A case is considered as any case fulfilling the case reference definition by Robert Koch Institute thus any laboratory confirmed toxigenic or *tox*-gene-positive diphtheria case or a case with an epidemiological link to a laboratory confirmed diphtheria case. [b] A classic respiratory clinal manifestation is any case with respiratory manifestation with pseudo-membrane, all other cases with respiratory manifestations are considered as mild. [c] Except for two cases with unknown sex (both *C. diphtheriae* cases in 2022-2023), all cases were female or male.[d] Age was unknown for two C. diphtheriae cases and two C. ulcerans cases, with one case of each species reported in 2018 and one in 2025. [e] The reported residence of each case at the time of notification was classified according to the Federal Institute for Research on Building, Urban Affairs and Spatial Development (BBSR) categories, distinguishing sparsely populated rural districts, more densely populated rural districts, urban districts (<100.000 inhabitants), small cities (100.000–500.000 inhabitants), and large cities (>500.000 inhabitants). [f]Travel associated refers to the case traveling abroad 21 days prior to infection, migration-associated if the case was reported to have migrated to Germany within 21 days prior to detection or stayed at the time of notification in an asylum reception centre, autochthonous if the person stayed in Germany 21 days prior to detection or unknown if no information was available. [g]We considered cases as fully vaccinated if at least 3 vaccination doses had been received, and partially vaccinated when less than three doses but at least one dose were received. Vaccinations were only taken into consideration if they occurred 30 days before disease onset or, if not available, date of notification

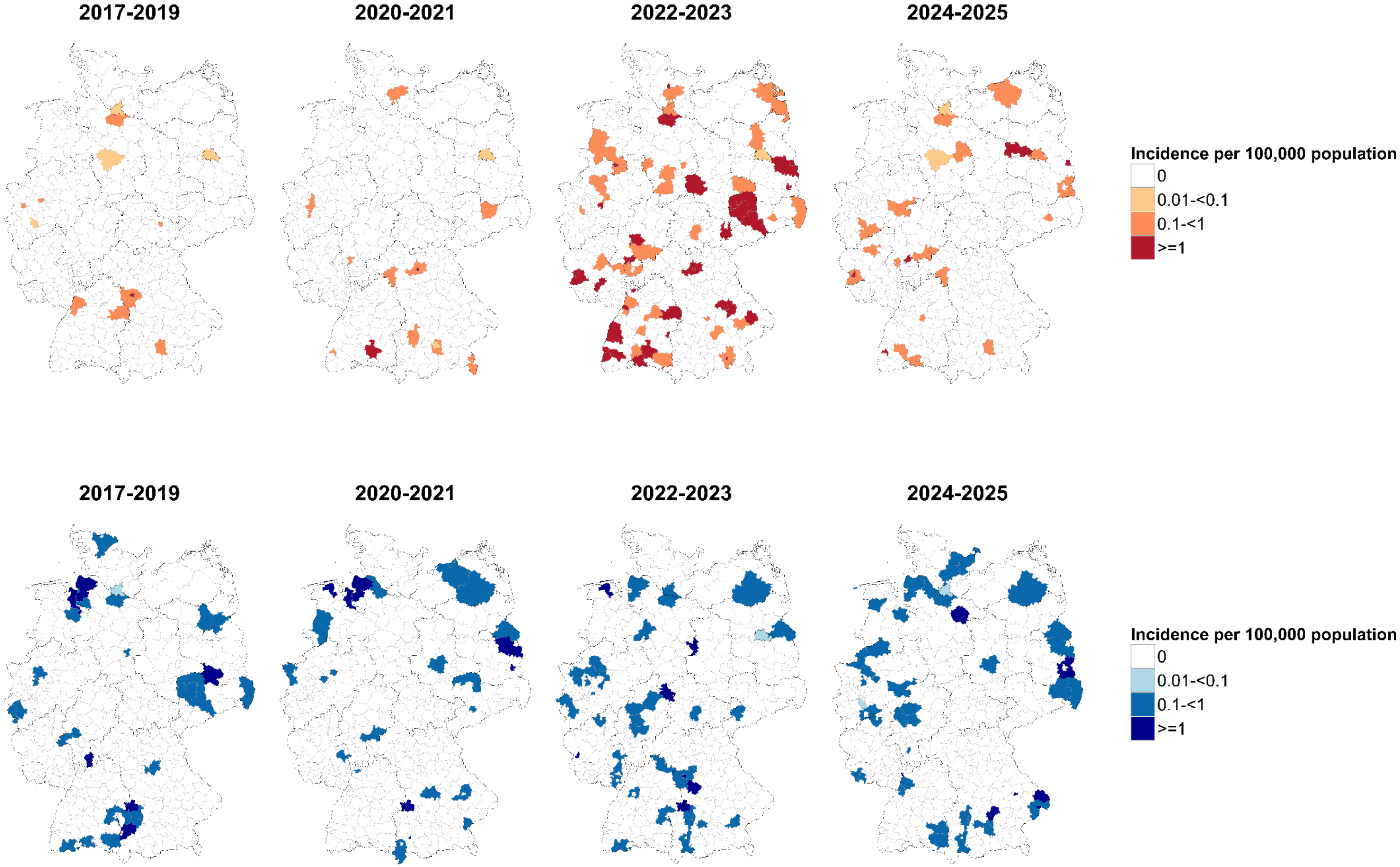

Supplementary Figure S1: ***C. diphtheriae*** **and** ***C. ulcerans*** **incidence per 100,000 inhabitants by district and phase, Germany, 2017–2025**

Supplementary Table S2: Deaths, hospitalizations and length of hospitalization among diphtheria cases by species and clinical manifestation, Germany, 2017 – 2025 (n=512)

	Overall		*C. diphtheriae*		*C. ulcerans*		*C. diphtheriae*				*C. ulcerans*			
	n	%	n	%	n	%	Classic respiratory	Mild respiratory	Cutaneous	Double manifestation	Classic respiratory	Mild respiratory	Cutaneous	Double manifestation
Total cases, n	512		343		163		7	15	306	18	2	6	154	4
Deaths due to the disease, n	5	1%	3	1%	2	1%	1	2	0	0	-	0	2	0
Cases with hospitalization data, n (%)	476	93%	316	91%	160	96%	7	15	276	18	2	6	148	4
Hospitalised cases, n (%)	136	28.6%	87	28%	49	31%	4	8	68	7	1	1	44	3
Hospitalised cases with hospital stay duration data, n (%)	111	81.6%	64	73.6%	47	95.9%	2	4	53	5	0	1	44	1
Median hospital stay length in days (Q1-Q3)	7 (3–10)		6 (1–10)		8 (6–10)		6 (4–8)	1 (1–2)	7 (3–10)	8 (2–15)	-	6 (6–6)	8 (6–10)	0 (0–0)

Supplementary Table S3: Number of co-infections in cutaneous *C. diphtheriae* and *C. ulcerans* cases included in the enhanced diphtheria surveillance project, 2017–2022 (n=101)

	C. diphtheriae	*C. ulcerans*
Total cases, n	74	27
**Co-infections**		
Cases with information on co-infections, n (%)	67	27
Cases with at least one co-infection	58	19
**Number of co-infections**		
1 co-infection agent	20	7
2 co-infection agents	17	7
3 co-infection agents	12	0
4 or more co-infection agents	2	2

Supplementary Table S4: All reported co-infection agents among cutaneous diphtheria cases reported through the enhanced diphtheria surveillance, 2017–2022

C. diphtheriae	***C. ulcerans***
Arcanobacterium haemolyticum	*Acinetobacter* spp.
Citrobacter koseri	*Bacteroides pyogenes*
Corynebacterium striatum	*Enterococcus faecalis*
Corynebacterium ulcerans	*Escherichia coli*
Finegoldia magna	*Helcococcus kunzii*
Leclercia adecarboxylata	Group G *Streptococcus*
Methicillin-resistant *Staphylococcus aureus* (MRSA)	*Klebsiella oxytoca*
Mycobacterium tuberculosis	*Klebsiella pneumoniae* spp.
Staphylococcus aureus	Methicillin-resistant *Staphylococcus aureus* (MRSA)
Staphylococcus epidermidis	*Morganella morganii* spp.
Staphylococcus spp.	*Peptoniphilus* spp.
Streptococcus agalactiae	Proteus mirabilis
Streptococcus dysgalactiae	*Pseudomonas aeruginosa*
Streptococcus pyogenes	*Pseudomonas* spp.
Streptococcus viridans	*Streptococcus dysgalactiae*
	Streptococcus pyogenes
	Streptococcus serogroup D
	Staphylococcus aureus

Supplementary Table S5: Wound locations in cutaneous *C. diphtheriae* and *C. ulcerans* cases included in the enhanced diphtheria surveillance project, 2017–2022 (n=101)

	C. diphtheriae	*C. ulcerans*
**Total cases, n**	74	27
**Wound locations**		
Cases with information on wound location, n (%)	28	17
Feet only	6	11
Lower legs	17	5
Legs	2	0
Genital	3	0
Feet and hands	1	0
Hands	0	1
